# Data on correlations between T cell subset frequencies and length of partial remission in type 1 diabetes

**DOI:** 10.1016/j.dib.2016.07.059

**Published:** 2016-08-06

**Authors:** Aditi Narsale, Rosita Moya, Hannah Kathryn Robertson, The Type 1Diabetes TrialNet Study Group, Joanna Davida Davies

**Affiliations:** San Diego Biomedical Research Institute, 10865 Road to the Cure, Suite 100, San Diego, CA 92121, USA

**Keywords:** Type 1 diabetes, T cell subsets, Partial remission

## Abstract

Partial remission in patients newly diagnosed with type 1 diabetes is a period of good glucose control that can last from several weeks to over a year. The clinical significance of the remission period is that patients might be more responsive to immunotherapy if treated within this period. This article provides clinical data that indicates the level of glucose control and insulin-secreting β-cell function of each patient in the study at baseline (within 3 months of diagnosis), and at 3, 6, 9, 12, 18 and 24 months post-baseline. The relative frequency of immune cell subsets in the PBMC of each patient and the association between the frequency of immune cell subsets measured and length of remission is also shown. These data support the findings reported in the accompanying publication, “A pilot study showing associations between frequency of CD4+ memory cell subsets at diagnosis and duration of partial remission in type 1 diabetes” (Moya et al., 2016) [1], where a full interpretation, including biological relevance of the study can be found.

**Specifications Table**

**Table:** 

Subject area	*Biology*
More specific subject area	*Immunology of patients newly diagnosed with type 1 diabetes*
Type of data	*Tables, Figures*
How data was acquired	*Flow Cytometry*
Data format	*Raw, analyzed*
Experimental factors	*Frozen peripheral blood mononuclear cells (PBMC) are thawed and stained with panels of monoclonal antibodies specific for markers that define T cell subsets.*
Experimental features	*The relative frequency of T cell subsets is determined by Flow Cytometry using an LSRFortessa for acquisition and FlowJo software for analysis.*
Data source location	*United States*
Data accessibility	*Data is provided within this article.*

**Value of the data**

• The data can be added to a larger patient data base to determine average length of partial remission and β-cell function over time in children diagnosed with type 1 diabetes between 9 and 16 years of age.
• Identifying a predictor at diagnosis that can predict length of remission in children with type 1 diabetes might be used to stratify and select patients most likely to respond to immunotherapy.
• These data introduce a multimetric approach that might be useful in predicting disease progression that might be applicable to a variety of disease states and stages including progression to type 1 diabetes, cancer progression, and responsiveness to immunotherapy.

## 1 Data

The data provided in this article show the IDAA1c (Supplementary Table 1A) and C-peptide levels (Supplementary Table 1B) for each patient in the study at baseline (within 3 months of diagnosis) and at 3, 6, 9, 12, 18 and 24 months post-baseline. Supplementary Table 2 shows the relative frequency of CD4^+^ CD45RO^+^ cells, activated Treg, and CD4^+^ CD25^+^ CD127^hi^ cells in PBMC at baseline and at 3 months post-baseline, stratifying patients into those with either good (Supplementary Table 2A) or poor glycemic control (Supplementary Table 2B). For patients with good glycemic control, the association between the frequency of each cell subset at baseline with length of time the HbA1c levels are maintained below 7% (Supplementary Fig. 1), and at 3 months with length of remission using IDAA1c (Supplementary Fig. 2) is shown.

## 2 Experimental design and materials and methods

### 2.1 Patient population

Archived peripheral blood mononuclear cells (PBMC) from 9 female and 10 male newly diagnosed type 1 diabetic patients were obtained from TrialNet Ancillary Studies. Patients were between 9 and 16 years of age and enrolled in the placebo groups of TrialNet clinical trials. Two PBMC samples from each patient were analyzed, one taken at baseline (within 3 months of diagnosis) and one 3 months later. The study was blinded. Clinical parameters were evaluated at baseline and again at 3, 6, 9, 12, 18, and 24 months post-baseline.

### 2.2 Measurement of partial remission and β-cell function using IDAA1c and C-peptide AUC

A standard formula, HbA1c (%)+(4×insulin dose (U/kg per 24 h), is used to take into account both insulin requirement and HbA1c levels in a single value, the Insulin Dose Adjusted A1c (IDAA1c) [2]. An IDAA1c equal to or less than 9 indicates the partial remission period [3]. Partial remission measured using IDAA1c is associated with a C-peptide AUC_120_ level of at least 108, equivalent to a mean C-peptide level of 0.9 ng/ml/min or 0.3 pmol/ml [2]. In this study the end of partial remission is between the last visit when IDAA1c is equal to or less than 9 and the first visit when IDAA1c is greater than 9. A more accurate estimate of time of end of partial remission was determined by interpolating between these two observed values. Length of remission is the time between diagnosis and end of remission. Stimulated C-peptide was measured in ng/ml and AUC calculated over 120 min using the trapezoidal rule, with observed C-peptide values at 0, 15, 30, 60, 90, and 120 min.

### 2.3 Type 1 diabetes PBMC analysis by Flow Cytometry

Vials of PBMC from patients with T1D were thawed and stained with each of the following panels of mAbs. Healthy subject PBMC were thawed and used as positive controls for mAb staining in each experiment. To identify memory (CD45RA^−^, CD45RO^+^) cells within the total CD4 compartment we used either APC-conjugated anti-CD4 (BioLegend, clone OKT4) with PerCP-Cy5-5-conjugated anti-CD3 (BioLegend, clone OKT3), PE-conjugated anti-CD45RO (BioLegend, clone UCHL1), and APC-H7-conjugated anti-CD45RA (BD Biosciences, clone HI100). To identify CD4^+^ CD25^+^ CD127^hi^ (CD25^+^ CD127^hi^) we used the BD Biosciences Treg cocktail (FITC-conjugated anti-CD4 (clone SH3), Alexa Fluor 647-conjugated anti-CD127 (clone HIL-7R-M21), and PE-Cy7-conjugated anti-CD25 (clone 2A3)), with PerCP-Cy5-5-conjugated anti-CD3. Activated Treg were identified using FITC-conjugated anti-CD4 (BioLegend, clone OKT4), APC-H7-conjugated anti-CD45RA and PerCP-Cy5.5-conjugated anti-Foxp3 (BD Biosciences, clone 236a/E7), using a standard published protocol used to identify activated Treg [4]. Data were acquired on an LSRFortessa. Isotype controls were used in every experiment and for every antigen-specific antibody. Additional CD4^+^ naïve and memory cell subsets were analyzed but none showed a significant correlation with length of partial remission [1].

### 2.4 Statistics

Associations of clinical variables with either length of time the HbA1c was maintained below 7%, or length of partial remission were assessed through Pearson correlations. All analyses were performed with Graphpad Prism.

## Footnotes

Appendix A Supplementary data associated with this article can be found in the online version at doi: 10.1016/j.dib.2016.07.059.
