# Supplementary material for: Data on correlations between T cell subset frequencies and length of partial remission in type 1 diabetes
Source: Data Brief. 2016 Aug 6;8:1348–51. doi: 10.1016/j.dib.2016.07.059 (PMC4992037; doi:10.1016/j.dib.2016.07.059)
Supplement: Supplementary file 2 — Supplementary material [file mmc2.zip › Supplementary Table 1.docx]

**Table 1**  IDAA1c and C-peptide AUC with time

A. IDAA1c

Patient #^1^ baseline^2^ IDAA1c at time post-baseline (months)^3^

0 3 6 9 12 18 24

1 9.0 9.8 9.5 9.9 10.2 10.9 12.4

2 10.1 10.2 13.0 12.4 13.1 NT^4^ NT

3 11.7 11.4 13.8 12.8 10.0 14.4 13.5

4 9.1 8.7 8.7 9.3 9.4 9.2 10.6

5 7.8 9.0 9.6 9.0 11.2 10.9 11.5

6 8.5 7.4 6.1 9.9 9.2 10.5 9.0

7 9.2 9.2 9.5 8.4 8.4 7.8 9.2

8 9.0 11.5 11.7 12.7 11.9 12.4 12.5

9 7.3 8.0 11.6 10.1 9.9 11.8 13.1

10 11.3 9.8 10.0 11.8 11.2 12.0 10.8

11 8.5 10.7 9.1 11.0 10.5 10.9 11.8

12 6.0 6.3 9.3 9.9 9.4 9.3 12.1

13 8.2 9.0 8.3 8.6 9.2 9.8 10.8

14 8.9 9.5 10.6 12.1 12.5 NT NT

15 7.2 7.4 8.3 12.3 11.2 13.0 11.8

16 9.2 8.4 8.8 NT 9.6 NT NT

17 7.8 8.3 9.2 10.0 10.0 NT 9.9

18 6.5 9.4 9.4 9.6 9.6 11.3 11.6

19 6.2 5.7 6.7 7.4 6.9 9.6 10.9

B. C-peptide AUC

Patient #^1^ Baseline^2^ C-peptide AUC at time post-baseline (months)^3^

0 3 6 9 12 18 24

1 232 157 105 103 54 44 19

2 382 202 150 228 147 NT NT

3 125 216 NT^4^ NT 148 159 77

4 280 324 327 306 217 171 NT

5 271 237 195 198 179 118 117

6 387 493 410 266 341 284 241

7 271 343 252 226 260 NT 218

8 127 17 5 3 3 3 0

9 314 429 17 346 215 NT 88

10 215 146 105 74 58 30 21

11 197 124 82 71 44 15 NT

12 354 326 297 141 168 136 NT

13 276 203 159 140 84 55 18

14 216 86 47 48 26 11 NT

15 264 283 265 150 136 86 149

16 213 140 NT NT NT NT NT

17 156 115 59 9 14 NT 0

18 196 196 151 NT 101 50 53

19 332 312 224 NT 208 207 NT

1. Patient numbers were randomly assigned by our group and are consistent throughout the manuscript.

2. Baseline measurements were taken within 3 months of diagnosis.

3. C-peptide AUC value at each time point post-baseline as indicated.

4. NT = not tested.
