# Supplementary material for: Data on correlations between T cell subset frequencies and length of partial remission in type 1 diabetes
Source: Data Brief. 2016 Aug 6;8:1348–51. doi: 10.1016/j.dib.2016.07.059 (PMC4992037; doi:10.1016/j.dib.2016.07.059)
Supplement: Supplementary file 2 — Supplementary material [file mmc2.zip › Supplementary Table 2.docx]

**Table 2**  Cell subset frequencies in PBMC samples collected at baseline and 3 months post-baseline from 19 T1D patients stratified by good and poor glycemic control

A. Good glycemic control

Patient ^1^ CD4^+^ CD45RO^+^ activated Treg CD25^+^ CD127^hi^

base^2^  3 mo^3^ base 3 mo base 3 mo

4 33.5 32.7 NT NT 3.2 4.52

5 38.6 31.7 NT 2.12 9.73 7.37

6 31.4 26.4 1.22 1.45 9 NT

7 57.9 43.9 2.09 3.7 25.2 30.2

9 38.1 33.1 NT NT NT 1.57

12 26.6 29.2 NT 0.68 3.72 4.62

13 50.3 39.7 1.37 0.65 14.8 14.3

15 38.7 41.1 NT 1.73 7.33 NT

16 32.9 40.3 0.81 NT 5.1 NT

17 46 33 0.81 1.13 9.57 5.4

18 27.6 26.4 1.3 0.96 4.44 3.36

19 47 37.1 1.41 1 11.8 6.51

B. Poor glycemic control

Patient ^1^ CD4^+^ CD45RO^+^ activated Treg CD25^+^ CD127^hi^

base^2^ 3 mo^3^ base 3 mo base 3 mo

1 38.7 39.1 NT 0.92 2.49 10.3

2 41.1 NT 2.4 NT 13 NT

3 63.8 55.7 1.33 NT 5.33 6.1

8 23.5 42.4 NT NT 2.92 11.7

10 38.5 38.3 1.37 1.67 7.81 12.4

11 22.3 25.3 0.76 NT 4.36 4.44

14 49.9 36.6 1 0.8 10.5 5.87

1. Patient numbers were randomly assigned by our group and are consistent throughout the manuscript.

2. Baseline cell subset frequencies (%) were tested within 3 months of diagnosis.

3. Cell subset frequencies (%) tested 3 months post-baseline.

4. NT = not tested.
