# Supplementary material for: Data on correlations between T cell subset frequencies and length of partial remission in type 1 diabetes
Source: Data Brief. 2016 Aug 6;8:1348–51. doi: 10.1016/j.dib.2016.07.059 (PMC4992037; doi:10.1016/j.dib.2016.07.059)
Supplement: Supplementary file 2 — Supplementary material [file mmc2.zip › Supplementary Figure Legend.docx]

**Figure Legends**

**Fig 1 The relative frequency of activated Treg cells correlates with length of time HbA1c levels are maintained below 7%** The correlation between the relative frequency of CD45RO^+^ cells (A), activated Treg cells (B), and CD25^+^ CD127^hi^ cells (C) at baseline and the length of time the HbA1c levels are maintained below 7%. Each symbol represents a patient. Pearson Correlation Coefficient (r), R squared (R^2^), and statistical significance (p) are shown on each panel.

**Fig 2 Correlations between the relative frequency of CD45RO^+^, activated Treg, and CD25^+^ CD127^hi^ cells with length of partial remission at 3 months post-baseline measured using IDAA1c** The correlation between the relative frequency of CD45RO^+^ cells (A), activated Treg cells (B), and CD25^+^ CD127^hi^ cells (C) at 3 months post-baseline and the length of partial remission measured using IDAA1c. Each symbol represents a patient. Pearson Correlation Coefficient (r), R squared (R^2^), and statistical significance (p) are shown on each panel.
