# Supplementary figures and images for: Data on correlations between T cell subset frequencies and length of partial remission in type 1 diabetes
Source: Data Brief. 2016 Aug 6;8:1348–51. doi: 10.1016/j.dib.2016.07.059 (PMC4992037; doi:10.1016/j.dib.2016.07.059)

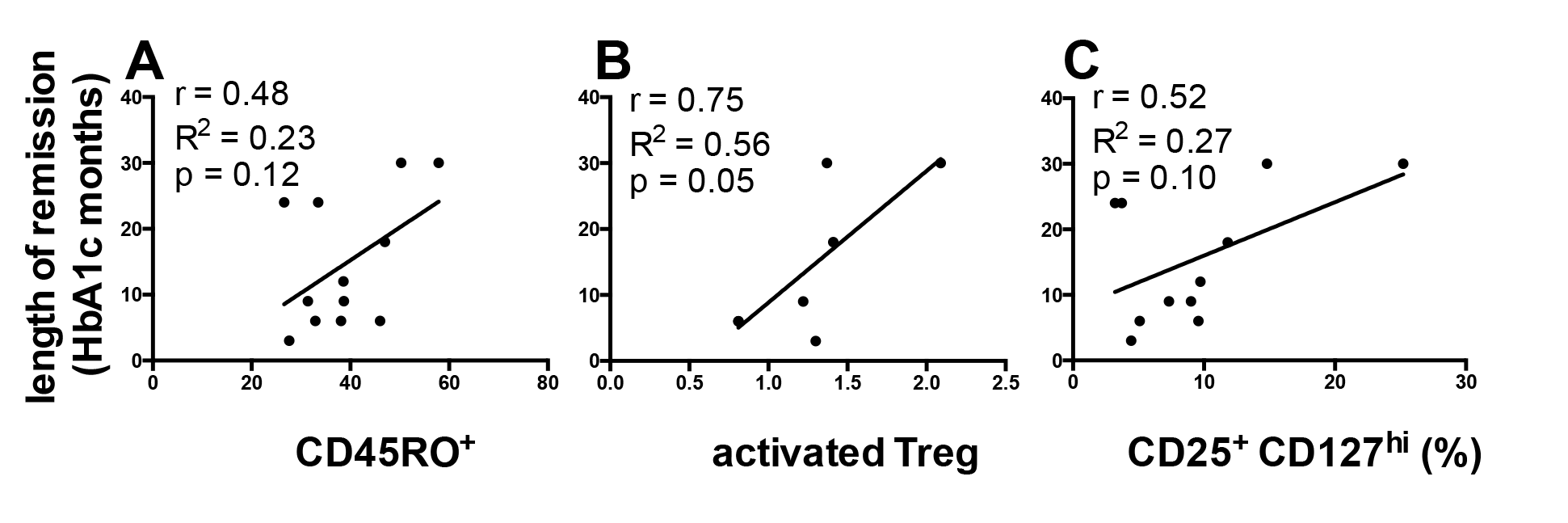

Supplement: Supplementary file 2 — Supplementary material [file mmc2.zip › Supplementary Figure 1.tif]

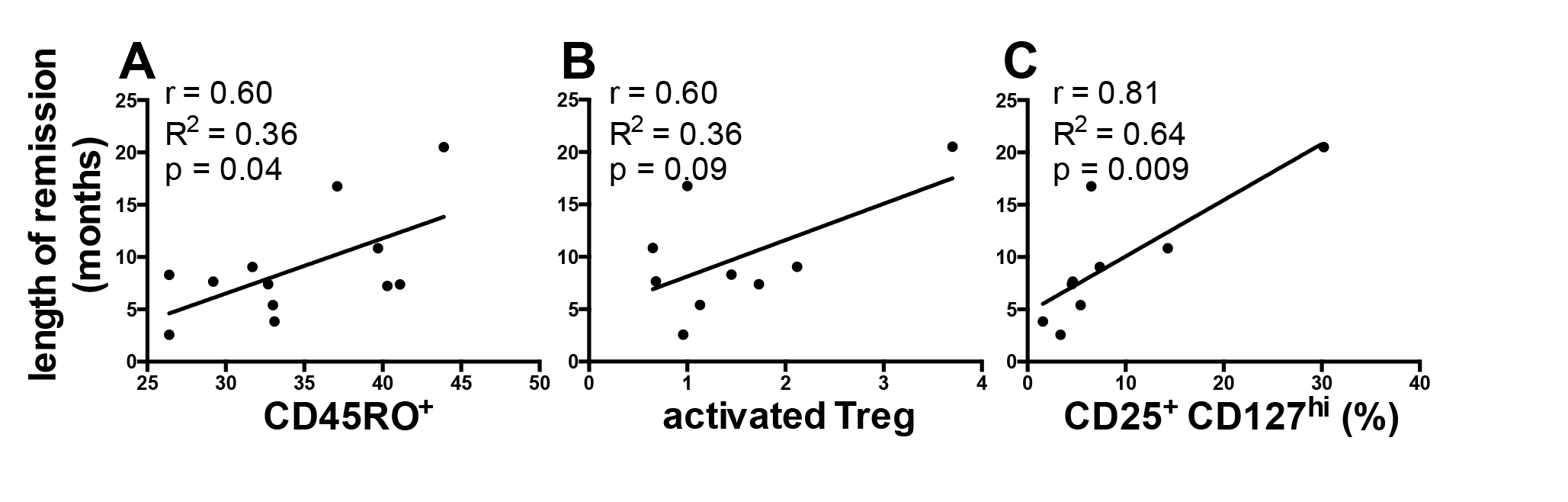

Supplement: Supplementary file 2 — Supplementary material [file mmc2.zip › Supplementary Figure 2.tif]
